# Urinary Tract Infections in Patients Undergoing Invasive Urodynamic Study: A Prospective Observational Study at a Tertiary Care Centre in Eastern India

**DOI:** 10.7759/cureus.52801

**Published:** 2024-01-23

**Authors:** Rohit Upadhyay, Khalid Mahmood, Rajesh K Tiwari, Ankit Raj

**Affiliations:** 1 Department of Urology, Indira Gandhi Institute of Medical Sciences, Patna, IND

**Keywords:** detrusor pressure, anti-biotic, urine culture, urinary tract infection, urodynamic studies

## Abstract

Objective: The aim of the study was to find the estimate of the prevalence of urinary tract infections following invasive urodynamic studies (UDS) in a hospital setup and to identify the risk factors related to it.

Method: A total of 100 patients were enrolled in this prospective observational study after standard preoperative work, which included both urine analysis and culture procedure. The study was carried out from April 2022 to April 2023 at the Department of Urology, Indira Gandhi Institute of Medical Sciences, India. Three days following the UDS test, all the patients underwent repeat urine analysis and culture, besides screening for any lower urinary tract symptoms, abdominal pain, and fever.

Result: Among all, 14 patients (i.e., 6.1% of 85 individuals) had significant bacteriuria, and six patients (4.7%) developed symptoms of UTI. However, a strong association was observed between the maximal detrusor pressure during voiding (Pdet at Q max) and post-void residue (PVR), which were >20 mL before UDS, along with positive urine cultures after UDS, which was significant at <0.05.

Conclusion: The study demonstrated that the risk of UTIs with this diagnostic technique is minimal and that prophylactic antibiotic medication is not necessary prior to UDS in all patients.

## Introduction

The practice of invasive urodynamic studies (UDS) is increasing day by day in clinical urology to pinpoint the diagnosis and pathophysiology behind urinary tract symptoms, especially in the lower side, and to pinpoint that bladder is an unreliable witness as it is believed that symptoms alone do not always correlate with signs and, hence, UDS is important [1].

Invasive UDS requires per urethral catheterization to fill the bladder with normal saline and record pressures during the filling and voiding phase. Ideally, before UDS, urine must be sterile [2]. However, after UDS, an incidence of 1.5-36% has been reported in the literature. Urinary tract infection (UTI) manifests as suprapubic pain, fever, hematuria, dysuria, increased frequency, anxiety, etc. [2,3]. Pyelonephritis risk is generally elevated, and there is a chance of future actual impairment in case, and UTI occurs after UDS [4]. The considerable variation in the incidence estimates of UTI after UDS may be caused by various factors, which include timing of urine testing, catheterization techniques, age differences between study populations, underlying health issues, UDS performance methods, and other definitions of UTI [5].

According to research, UTI has a significant financial impact on the entire world, costing an estimated 1.6 billion US dollars annually [6-8]. Pre-procedural antibiotics are frequently administered; however, it is unclear whether this should be done consistently or only in certain circumstances [9]. The majority of research advised regular prophylactic antibiotics for populations where the incidence of UTI is more than 10% [10]. Prophylaxis primarily benefits patients undergoing invasive UDS by lowering the risk of serious infection problems caused by bacteriuria [10]. There were some common measures to prevent UTI after UDS that involved safe pre-procedural urine culture screening, cleaning of antimicrobial urethral meatal, and use of prophylactic antibiotics [2]. Prophylactic antibiotics safely reduced rates of significant bacteriuria after UDS, but the effect on symptomatic UTI is unknown [11].

Regularly administering preventive antibiotics can have negative consequences and can encourage the growth of infections that are resistant to antibiotics [2]. The literature has identified many risk factors, including advanced pelvic organ prolapse, higher BMI, older age, hypothyroidism, diabetes, multiparous women, advanced age, and past surgery for UTI and incontinence therapy prior to UDS investigation [12]. The rationale of this study was to identify those individuals who were at high risk of UTI and to estimate the prevalence of UTI subsequent to UDS in the local community.

## Materials and methods

Before the initiation of the study, all the patients were explained about the nature of the study that the confidentiality of the participants will be protected at all levels and that they can withdraw from the study at any moment. Then, informed consent was obtained from the patients in their own language, and a detailed history, along with a physical examination, was done and documented in the proforma.

Study site: The study was carried out from April 2022 to April 2023 at the Department of Urology at Indira Gandhi Institute of Medical Sciences, India.

Ethical approval: Ethical approval to conduct the study has been granted by the Ethics Committee of Indira Gandhi Institute of Medical Sciences, India, under letter no. 514/IEC/IGIMS/2022.

Data collection: Demographics such as age, gender, occupation, any other previous history of surgical procedure, comorbidities, along with BMI, were documented. CBC, KFT, RBS, urine culture sensitivity report, and previous radiological investigations were recorded. A urine dipstick method test was carried out before UDS in all patients immediately. After that, patients were subjected to urodynamic evaluation using the UROCOMP 2000 machine (Status Medical Equipment, Maharashtra, India). Then, prior to catheterization and initiation of UDS, the uroflowmetry test was done for all patients, and the maximum flow rate (Q max) and PVR were documented.

Study procedure: The bladder was catheterized with two 6 Fr infant feeding tubes under sterile conditions, among which one was for measurement of vesical pressure and the other one was for bladder filling. The rectal catheter 10 F was inserted via the anus using lubricants, with the tip positioned 5-10 cm above the anal margin. Upon drying the perianal region, the catheter was taped near the anal margin as feasible. Filling cytometry was done at 20 mL/minute by using normal saline at room temperature with the patients in the lithotomy position. Voiding was performed at the same position when capacity was achieved.

During bladder filling, bladder sensation included the first sensation of filling, and either normal or strong desire to void, urgency or pain, detrusor activities, bladder compliance, bladder capacity, and leak point pressure were recorded. At the time of voiding, the voided volume, Q max, the average flow rate, the Pdet on maximum flow, and residual urine after the voiding phase were recorded.

All patients were instructed to take fluid in increased amounts after the procedure and to record any event of fever, pain, or lower urinary tract symptoms (LUTS) over the following seven days. Asymptomatic bacteriuria was defined as colony count >=10^4^ CFU/mL without any signs or symptoms. Significant bacteriuria was defined as colony count >10^1^ CFU/mL or CFU <10^3^ CFU/mL with symptoms and signs, such as fever, urinary urgency or frequency, dysuria, suprapubic or flank pain, etc. Pyuria was defined as microscopic evidence of more than or equal to 5-10 WBC per high power field. The urine culture report of all the patients was analyzed and documented.

Study population: A total of 100 consecutive patients undergoing invasive UDS were enrolled based on inclusion criteria. The study participants were recruited as per the following inclusion criteria, which included patients >18 years of age, patients undergoing an invasive UDS performed at IGIMS, Patna, during the study period, and patients who can provide consent to participate in the study. Participants were excluded on the basis of the following criteria: patients with a history of recurrent UTIs, patients who are on the long-term periurethral or suprapubic catheter or clean intermittent catheterization, patients with positive urine culture reports or symptoms of UTI or positive urine dipstick test for nitrites or leukocytes on the day of the procedure, and patients who were lost to follow-up.

Statistical analysis: Based on previous literature, we calculated the sample size using the formula, n^1^ = (σ^2^ + σ^2^ / K) (Z1 - α/ 2 + Z1 - β)/ d^2^, at 95% power and 5% type-2 error; n^2^ = K * n^1^, where d = difference between 2 means; σ^2^ = variance; K = ratio of the sample size for groups 1, 2, and 3; α = probability of type-1 error; β = probability of type-2 error; and Z = critical value given for α and β.

The information was gathered in an Excel spreadsheet. For performing statistical tests, Statistical Product and Service Solutions (SPSS, version 21.0 (trial version); IBM SPSS Statistics for Windows, Armonk, NY) was used. Data may contain both continuous and categorical variables. Therefore, quantitative variables were expressed as mean ± standard deviation (SD). For continuous variables such as age, data were displayed as mean and standard deviation, and for categorical variables, such as gender and profession, data were expressed as frequency and percentage. Appropriate statistical tests were applied for the generation of results and inferences. The concentration of detrusor pressure was evaluated by a one-way ANOVA test to analyze the variance within the groups, and Tukey’s test was used for post-hoc analysis. For all statistical tests, a two-sided p-value < 0.05 was considered as the level of significance. T-test and chi-square test were used to evaluate the mean and SD and p-value, respectively, in the results of several categorical data.

## Results

**Figure 1:**
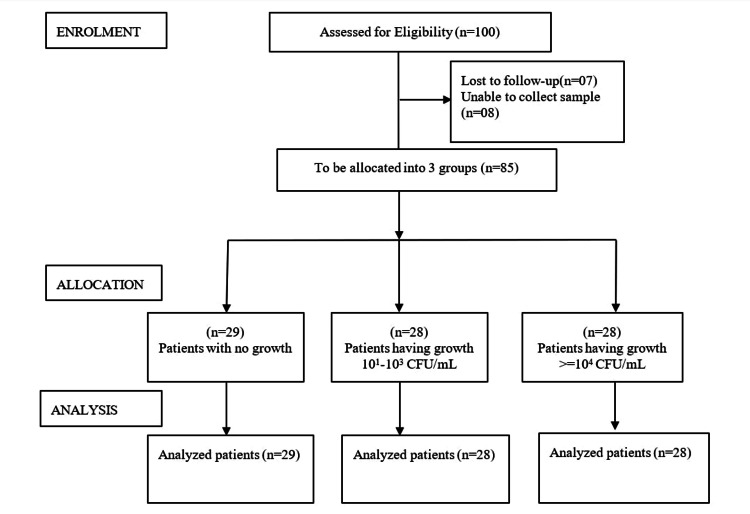
CONSORT diagram describing the enrolment, allocation, and analysis of participants

Out of the 100 individuals who initially signed up for the trial, 15 people were later dropped out due to the lack of follow-up and failure to take a urine culture following UDS. Ultimately, 85 patients completed the study. Patients were allocated to three separate study groups depending upon colonies formed in urine culture; for example, 29 patients were in the group of no growth, 28 patients were present in the group of patients having 101-103 CFU/mL, and other 28 in patients with > = 104 CFU/mL. The details of enrolment, allocation, and analysis of the included participants are provided in the CONSORT diagram (Figure 1) [13].

Table 1 demonstrates the baseline characteristics of the study population. Participants had a mean age of 52.4±10.2 in the no-growth group and a mean BMI of 28.8±5.5 kg/m^2^ in the same group, whereas participants having high colonies formed had a mean age of 53.8±13.8 and a BMI of 26.3±2.2.

**Table 1:** Characteristics of study population Data are presented as mean ± SD p-value is significant at < 0.05.

Characteristics	No growth (n=29)	10^1^-10^3 ^CFU/mL (n=28)	>=10^4 ^CFU/mL (n=28)
Age (in years)	52.4±10.2	56.8±13.6	53.8±13.8
BMI (in kg/m^2^)	28.8±5.5	27.6±4.7	26.3±2.2
Height (in cm)	171.2±9.57	155.46±7.07	154.84±6.23
Weight (in kg)	70.33±14.01	65.32±10.24	67.96±10.31

The ANOVA test was used for the comparison of detrusor pressure in different groups of urine culture and simultaneously Tukey’s test was used for post-hoc analysis. The details of the concentration of detrusor pressure have been included in Table 2.

**Table 2:** Concentration of detrusor pressure in different colonies a: p-value=0.30 vs negative culture; b: p-value=0.02 vs negative culture Data are presented as mean±SD *p-value is significant as <0.05 (two-tailed).

Parameter	No growth	10^1^-10^3 ^CFU/mL	>=10^4 ^CFU/mL
Detrusor pressure at the maximum	42.5±23.5	57.0±23.3^a^	68.3±55.5^b^

One-way ANOVA revealed that patients with considerable bacteriuria had higher mean maximum pressures in their detrusors during urination than those with negative cultures (p-value=0.02), as compared with patients having considerable bacteriuria vs negative culture (p-value=0.30) (Table 3).

**Table 3:** Results of several categorical data Data are presented as mean±SD p-value is significant at <0.05. t-test was used to obtain the mean and standard deviation, and a chi-square test was used to obtain the p-value within the groups.

Variable	No growth (n=29)	10^1^-10^3 ^CFU/mL (n=28)	>=10^4 ^CFU/mL (n=28)	P-Value
Average flow rate before UDS	7.7±4.0	6.8±3.7	7.6±4.03	0.67
Maximum flow rate before UDS	19.6±11.5	19.6±3.4	20.2±4.03	0.64
Volume at a normal desire	224.1±80.8	242±142.8	253.3±84.7	0.56
Volume at strong desire	389.8±120	434.3±174.5	409.7±73.3	0.70
Volume at first desire	116.2±52.6	135.7±52.8	100±52.4	0.51
Average flow rate after UDS	7.5±3.6	8.2±2.2	6.3±3.1	0.61
Maximum flow rate after UDS	19.7±13.6	20.2±6	17.0±9.0	0.86

Out of 85 patients, six patients had symptomatic, culture-positive UTI, while others had asymptomatic culture-positive bacteriuria. Other than this, urinary leakage affected 81 individuals in all (56.1%). Six patients (4.7%) developed UTIs, and eight (6.1%) had severe bacteriuria. People with high levels of bacteriuria had no symptoms. Two patients developed hematuria and dysuria affecting five individuals with >105 colony count in U/C. All the patients who presented with post-UDS urinary tract infections were treated according to the culture and sensitivity report.

Twenty-one individuals with negative cultures experienced frequent urination and dysuria, which spontaneously improved with fluid intake. Urinary cultures and the abnormal initial PVR (pre-test) had a significant connection, according to the chi-square test (p<0.05). After UDS, patients with initial PVRs greater than 20 mL frequently developed bacteriuria or UTIs.

## Discussion

Six out of 85 trial participants experienced UTI (>=104 CFU/mL). Eight individuals showed high levels of asymptomatic bacteriuria. On the other hand, 21 symptomatic individuals had negative cultures. After giving adequate fluids, these individuals became asymptomatic. It appears that invasive urodynamic testing has a low risk of UTI, and monitoring for UTI was not necessary after UDS. Additionally, prophylactic antibiotic therapy is not required in all cases. In this study, all the patients who developed LUTS post-invasive urodynamic testing did not have UTI. Moreover, a urine routine and culture were mandatory before starting any antibiotics.

It has been observed in review research by Dass et al. that prophylactic antibiotics appear to lower the probability of bacteriuria following urodynamic investigations [12]; still, there was insufficient data to conclude that this impact also resulted in a decrease in symptomatic UTI. In this study, all the patients who had an initial post-void residual (PVR) of more than 20 ml had significant bacteriuria or UTI following UDS. According to Tsai et al., PVR volume >100 mL is a major risk factor for UTI in the urodynamic guideline, while antibiotic treatment is optional with just one risk factor [13]. A study by Sung-II Hwang et al. found that individuals with high PVR had a greater likelihood of developing a UTI [14]. On the other hand, our research shows no association between UTI and aberrant PVR (>20 mL) following UDS. The study also discovered a significant difference in PVR, which is 4.0 mL versus 67.1 mL between the pre- and post-UDS periods, respectively; however, this increase in PVR during the process of UDS was expected by considering the usage of catheters and other adjustments. In research by Ghanbari et al., it has been shown that prophylactic antibiotic therapy is only appropriate in case of PVR greater than 50 mL and possibly of high detrusor pressure [15].

According to Nicoll et al., UTI was diagnosed in 7.2% of patients with stress urinary incontinence (SUI) in UDS, whereas it was only 1.5% of individuals without urine leakage [16]. They also reported the significance of urethral obstruction in the emergence of UTI following UDS. Quek et al. stated that a lower average flow rate was listed as a risk factor for UTI following UDS, although the current investigation did not observe this difference [17].

It has been observed that diabetes and severe bacteriuria following UDS were significantly correlated [18]. The substantial increase in bacteriuria in diabetes individuals may be brought on by urethral catheterization. According to Tsai et al., it has been observed that patients with risk factors for UTI following UDS who have more than three normal vaginal deliveries, have UTI before the procedure, and have a low average flow rate (7 mL/second) should take prophylactic antibiotics after the procedure [13].

Due to the high risk of UTI that occurs after UDS, Pannek et al. recommended that antibiotic prophylaxis be used [19], while in another study, Bothig et al. found that prophylactic antibiotics are necessary when bacteriuria and reflex voiding occur prior to UDS [20]. In a meta-analysis study performed by Latthe et al., it was concluded that prophylactic antibiotics should be administered to 13 individuals undergoing UDS to prevent one UTI [11]. In 2017, the Society of Urodynamics, Female Pelvic Medicine, and Urogenital Reconstruction (SUFU) released a statement stating that all patients should be screened for UTI and, if the physician suspects a UTI, should first undergo a dipstick urinalysis before undergoing an ultrasound of the genitalia (UDS) [21]. According to research, it has been observed that adherence to EAU guidelines on antibiotic prophylaxis reduced the usage of antibiotics and lowered the prevalence of uropathogens [22]. Other than this, according to the EAU guidelines, two meta-analyses found no evidence of the benefits of antibiotic prophylaxis in reducing the rate of clinical UTI; however, the rate of bacteriuria was reduced [23]. Thus, the supporting statement of our research is that antibiotic prophylaxis seems unnecessary. However, to explain this contradiction, more research is necessary.

Limitations

The drawbacks of the study include a small sample size and further assessment of the process, which was difficult due to the unpredictability of the traditional sampling procedure.

## Conclusions

To conclude, the study showed that a smaller number of patients had urinary tract infections following invasive UDS. Participants with considerable bacteriuria had higher pressures in their detrusors. Other than this, as per research, antibiotic prophylaxis appears to be unnecessary. It has been observed that PVR was more than 20 mL prior to performing UDS, and significant detrusor pressure was present during voiding. A strong association was observed between urinary cultures and abnormal initial PVR.
